# FAST tuberculosis transmission control strategy speeds the start of tuberculosis treatment at a general hospital in Lima, Peru

**DOI:** 10.1017/ice.2021.422

**Published:** 2021-10-06

**Authors:** Dylan B. Tierney, Eli Orvis, Ruvandhi R. Nathavitharana, Shelley Hurwitz, Karen Tintaya, Dante Vargas, Patricia Segura, Silvana de la Gala, Leonid Lecca, Carole D. Mitnick, Edward A. Nardell

**Affiliations:** 1 Brigham and Women’s Hospital, Boston, Massachusetts, United States; 2 Harvard Medical School, Boston, Massachusetts, United States; 3 Analysis Group, Boston, Massachusetts, United States; 4 Beth Israel Deaconess Medical Center, Boston, Massachusetts, United States; 5 Socios En Salud Sucursal Peru, Lima, Peru; 6 Hospital Nacional Hipolito Unanue, Lima, Peru; 7 Department of Global Health and Social Medicine, Harvard Medical School, Boston, Massachusetts, United States

## Abstract

**Objective:**

To evaluate the effect of the FAST (Find cases Actively, Separate safely, Treat effectively) strategy on time to tuberculosis diagnosis and treatment for patients at a general hospital in a tuberculosis-endemic setting.

**Design:**

Prospective cohort study with historical controls.

**Participants:**

Patients diagnosed with pulmonary tuberculosis during hospitalization at Hospital Nacional Hipolito Unanue in Lima, Peru.

**Methods:**

The FAST strategy was implemented from July 24, 2016, to December 31, 2019. We compared the proportion of patients with drug susceptibility testing and tuberculosis treatment during FAST to the 6-month period prior to FAST. Times to diagnosis and tuberculosis treatment were also compared using Kaplan-Meier plots and Cox regressions.

**Results:**

We analyzed 75 patients diagnosed with pulmonary tuberculosis through FAST. The historical cohort comprised 76 patients. More FAST patients underwent drug susceptibility testing (98.7% vs 57.8%; OR, 53.8; *P* < .001), which led to the diagnosis of drug-resistant tuberculosis in 18 (24.3%) of 74 of the prospective cohort and 4 (9%) of 44 of the historical cohort (OR, 3.2; *P* = .03). Overall, 55 FAST patients (73.3%) started tuberculosis treatment during hospitalization compared to 39 (51.3%) controls (OR, 2.44; *P* = .012). FAST reduced the time from hospital admission to the start of TB treatment (HR, 2.11; 95% CI, 1.39–3.21; *P* < .001).

**Conclusions:**

Using the FAST strategy improved the diagnosis of drug-resistant tuberculosis and the likelihood and speed of starting treatment among patients with pulmonary tuberculosis at a general hospital in a tuberculosis-endemic setting. In these settings, the FAST strategy should be considered to reduce tuberculosis transmission while simultaneously improving the quality of care.

Tuberculosis (TB) continues to be an epidemic of major global health significance; ~10 million people develop the disease annually.^1^ Hospitals in TB-endemic settings are transmission hotspots^2^ and give rise to community outbreaks, particularly with drug-resistant (DR) strains.^3–5^ Cross-sectional surveys of hospitalized patients in TB-endemic settings have demonstrated a high proportion of patients with undiagnosed and/or untreated TB.^6–8^ These individuals can be hospitalized for extended periods in crowded wards, where TB is passed to other patients, healthcare workers, and visitors.^9–11^ Missed opportunities for case finding,^12^ slow diagnostic testing,^13,14^ and delayed treatment^15^ create conditions that allow nosocomial TB transmission to occur. Poor deployment of rapid drug-susceptibility testing (DST) aggravates the situation, precluding timely initiation of effective therapy for drug-resistant TB (DR-TB).^16^

Awareness is growing about the need to improve TB screening, diagnosis, and treatment to achieve TB transmission control in the hospital.^17,18^ Modeling reveals that decreasing exposure to patients with infectious TB through improved case detection and delivery of care will reduce transmission.^19^ The FAST (Find cases Actively, Separate safely, Treat effectively) strategy has been developed to speed the delivery of key elements of TB care to reduce hospital transmission of TB from patients with undiagnosed and/or untreated disease.^20,21^

The first step of FAST is universal active TB symptom and risk-factor screening of hospitalized patients. Those with symptoms or risk factors undergo rapid diagnosis and DST using nucleic acid amplification tests followed by the expedited start of effective treatment. Whether treatment is effective and stops transmission depends in large part on the match between the regimen and the susceptibility of the infecting strains of *Mycobacterium tuberculosis*, making the inclusion of rapid DST a critical component of FAST.^22^ Evidence suggests that even a few days of effective treatment may render a patient noninfectious.^22–24^ This impact contrasts starkly with the weeks of therapy often required to effect sputum smear and culture conversion, which has historically been used to signal the end of the infectious period.^25,26^

Interrupting hospital TB transmission through FAST hinges on the strategy’s ability to decrease the institutional exposure time to patients with untreated or inadequately treated TB. Using ‘time to’ process indicators, pilot studies performed in chest and TB hospitals have shown that FAST can shorten the time to TB diagnosis and effective treatment.^20,27^ In those settings, all patients have respiratory symptoms or a TB diagnosis on presentation. Here, we implemented FAST among a broader population of patients in a general hospital, and we examined its effect on time to TB diagnosis, DST, and treatment for patients with pulmonary TB in a single-center prospective cohort study with historical controls.

## Methods

### Setting

We implemented FAST at Hospital Nacional Hipolito Unanue (HNHU), a 700-bed general hospital in Lima, Peru, between January 1, 2016, and December 31, 2019. Peru has an annual TB incidence of 119 cases per 100,000 people, and the highest MDR-TB incidence in Latin America.^1^

Prior to the study period, HNHU relied on passive case finding to detect TB among hospitalized patients. Entry into the TB care cascade was driven primarily by patient self-report of TB symptoms during clinical encounters. Patients were then evaluated for pulmonary TB with 2 sputum samples tested by smear microscopy; in some patients with positive smears, GenoType (Hain Lifescience GmbH, Nehren, Germany) was also performed. Patients with 2 negative smears but clinical concern for TB were tested with mycobacterial culture on third sputum sample. HNHU clinicians from the National TB Program bore responsibility for treatment initiation according to national guidelines.

### FAST intervention

Trained FAST assistants approached and solicited information about cough and risk factors for pulmonary TB from all adult patients upon facility admission, regardless of presenting complaint. Those who screened positive were offered enrollment in the study. At least 2 sputum samples were tested for TB at HNHU. Methods used were smear microscopy, Xpert MTB/RIF (Xpert, Cepheid, Sunnyvale, CA) and/or GenoType MTBDRplus line probe assay. Mycobacterial culture and conventional DST were also performed. FAST assistants collected and transferred specimens to the HNHU laboratory and communicated diagnostic test results to clinicians. Patients who were diagnosed with TB were referred to hospital-based National TB Program staff, who were responsible for starting TB treatment as recommended by the Peru TB guidelines (as in the historical period).

The research study was conducted using a formative evaluation approach, with FAST process outputs guiding iterative changes in the intervention during the study period.^28^ In phase 1, from August 3, 2016, to June 19, 2017, screening questions were limited to cough for >2 weeks. Because this symptom was rarely reported, cough of any duration became the screening question in the second implementation phase (June 20, 2017, to January 1, 2018). Xpert testing was also introduced as part of the FAST protocol in this second phase. The final iteration of the FAST protocol was implemented on January 2, 2018, and continued through study end on December 31, 2019. It permitted inclusion based on any of the following criteria: cough of any duration (self-reported or observed by a healthcare provider during hospitalization), history of active TB, current active TB diagnosis and treatment, or contact of someone with active TB.

### Study population

**Fig. 1.**
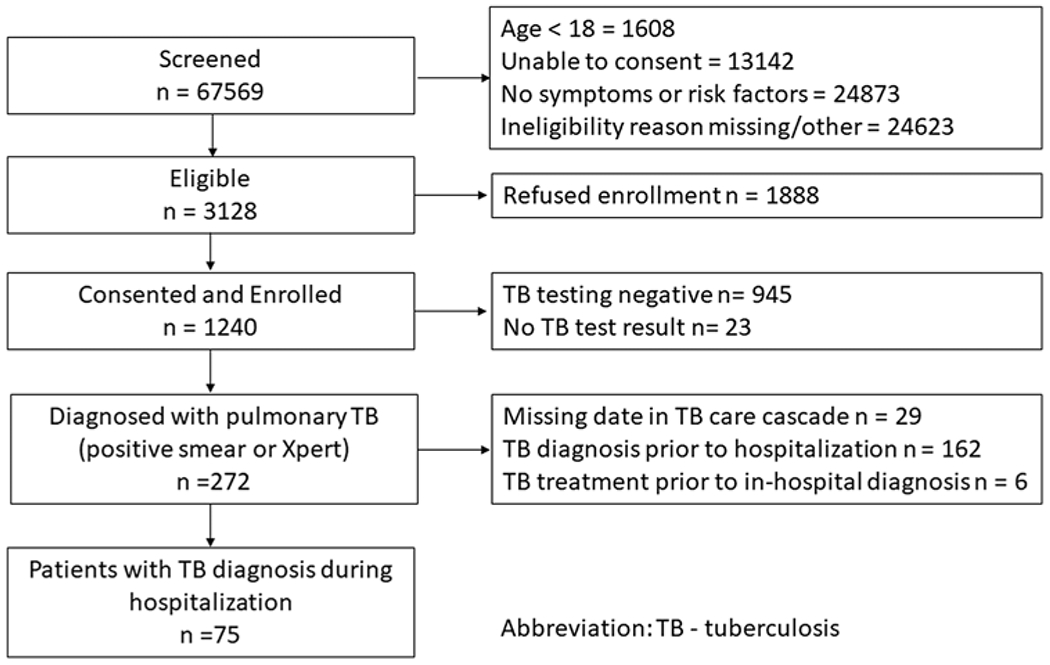

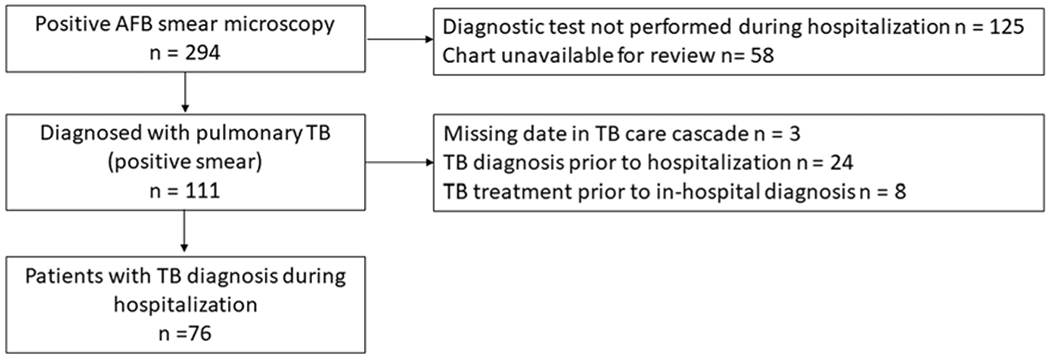
(A) Flowchart for prospective FAST cohort at Hospital Nacional Hipolito Unanue in Lima, Peru. Note. TB, tuberculosis. (B) Flowchart for historical cohort at Hospital Nacional Hipolito Unanue in Lima, Peru. Note. AFB, acid-fast bacilli; TB, tuberculosis.

We prospectively enrolled consenting patients with a laboratory-confirmed diagnosis of pulmonary TB between August 3, 2016, and December 31, 2019 (Fig. 1a). This period was established to permit expected enrollment of a sample of healthcare workers to analyze the impact of the FAST strategy on TB infection. The comparative historical cohort included all patients with laboratory-confirmed pulmonary TB diagnosed at HNHU between January 1, 2016, and July 23, 2016, a period immediately prior to FAST implementation (Fig. 1b). Records for patients hospitalized prior to 2016 were not available for review.

### Data collection and outcome definitions

We collected demographic variables and results from TB diagnostic tests, dates of hospitalization and discharge, and key dates from the TB care cascade (Supplementary Material 1 online). Date of TB diagnosis was defined as the date of the first positive smear microscopy or positive Xpert result for either of the first 2 sputum samples obtained. Date of DST was defined as the date of Xpert, GenoType, or culture-based DST result, whichever was available first. TB treatment initiation date was defined as the date TB treatment was started according to the patient’s medical record. We made a distinction between treatment and ‘indicated’ treatment; the latter describes the TB treatment regimen recommended by national guidelines using DST results. In patients with drug-susceptible TB (DS-TB), indicated treatment was defined as the standard World Health Organization first-line regimen.^29^ For patients with rifampin-resistant (RR) or multidrug-resistant TB (MDR-TB), indicated treatment contained at least a fluoroquinolone and an aminoglycoside or polypeptide if there was no documented resistance to these agents.

### Analysis

We calculated the proportion of patients diagnosed using DST and treated (with and without an indicated regimen). We compared these proportions between historical and prospective cohorts. We also calculated the days from hospital admission to TB diagnosis and from TB diagnosis to initiation of TB treatment and indicated treatment. The sum of these periods was days from hospital admission to (indicated) TB treatment initiation. Length of stay was the period between hospital admission and hospital discharge.

The analysis excluded patients who were missing date of hospitalization, TB diagnosis, or discharge (Fig. 1a and 1b). Patients were also excluded from the analysis if the diagnosis of their current episode of TB predated hospitalization at HNHU. Patients who received empiric TB treatment prior to diagnosis (ie, TB treatment initiation date preceding the date of TB diagnosis) were excluded from the primary analyses.

Odds ratios were used to compare proportions and median time (in days) to each event in the TB care cascade for historical and prospective cohorts. Time to event was also compared with Kaplan-Meier plots and Cox regressions. Follow-up time was censored at discharge date in patients who had not experienced the event prior to discharge. Unadjusted hazard ratios (HRs) and 95% confidence intervals (CIs) were reported; values >1.0 indicate shorter time to and higher likelihood of the event for the prospective cohort. The proportion of participants who started treatment within 2 days of diagnosis, along with the 95% CI, was also reported.

The ethics boards at HNHU and Brigham and Women’s Hospital approved the study.

## Results

We prospectively screened 67,569 hospitalized patients from August 3, 2016, to December 31, 2019, and identified 3,128 (4.6%) with cough or other TB risk factors who were eligible to participate in the FAST study (Fig. 1a). We consented and enrolled 1,240 individuals, 272 of whom had at least 1 positive sputum test for TB. Of those, 75 were diagnosed with laboratory-confirmed TB during hospitalization. Three patients were diagnosed in phase 1 of FAST implementation, 16 were diagnosed in phase 2, and 56 were diagnosed in phase 3. An additional 162 sputum-positive patients (60%) had a TB diagnosis that predated hospitalization. In the historical period, we identified 76 patients with TB newly diagnosed during hospitalization (Fig. 1b).

In the prospective FAST cohort, Xpert testing was performed on samples collected from 68 (90.7%) patients. Results were positive in 67 (98.5%), including 26 who had negative microscopy results (Table 1). All patients in the historical cohort had positive smear microscopy; none were tested by Xpert.

Compared with historical controls, more FAST patients (98.7% vs 57.8%; OR, 53.8; 95% CI, 7.1–407.74; *P* < .001) had DST results during hospitalization. Among those with DST, MDR-TB was detected in 18 (24.3%) of 74 of the prospective cohort and 4 (9%) of 44 of the historical cohort (OR, 3.2; 95% CI, 1.01–10.2; *P* = .03). In addition, 2 patients in the prospective cohort had isoniazid mono-resistance and 2 had rifampin mono-resistance.

The times from hospital admission to TB diagnosis were similar between the prospective and historical cohorts (HR, 0.88; 95% CI, 0.63–1.22; *P* = .46) (Table 2). The time from admission to diagnosis including DST, however, was shorter during the FAST period (HR, 23.71; 95% CI, 10.39–51.11; *P* < .001).

Under the FAST strategy, 54 patients (72%) with a new diagnosis of pulmonary TB began treatment during hospitalization compared to 39 (51.3%) in the historical cohort (OR, 2.44; 95% CI, 1.23–4.79; *P* = .012). Less time elapsed between TB diagnosis and initiation of TB treatment in the FAST cohort than in the historical cohort (HR, 2.54; 95% CI, 1.66–3.89; *P* < .001). The estimated proportion receiving TB treatment within 2 days of diagnosis was 75% (95% CI, 0.64–0.86) in the FAST cohort compared to 30% (95% CI, 0.2–0.43) in the historical cohort.

**Fig. 2.**
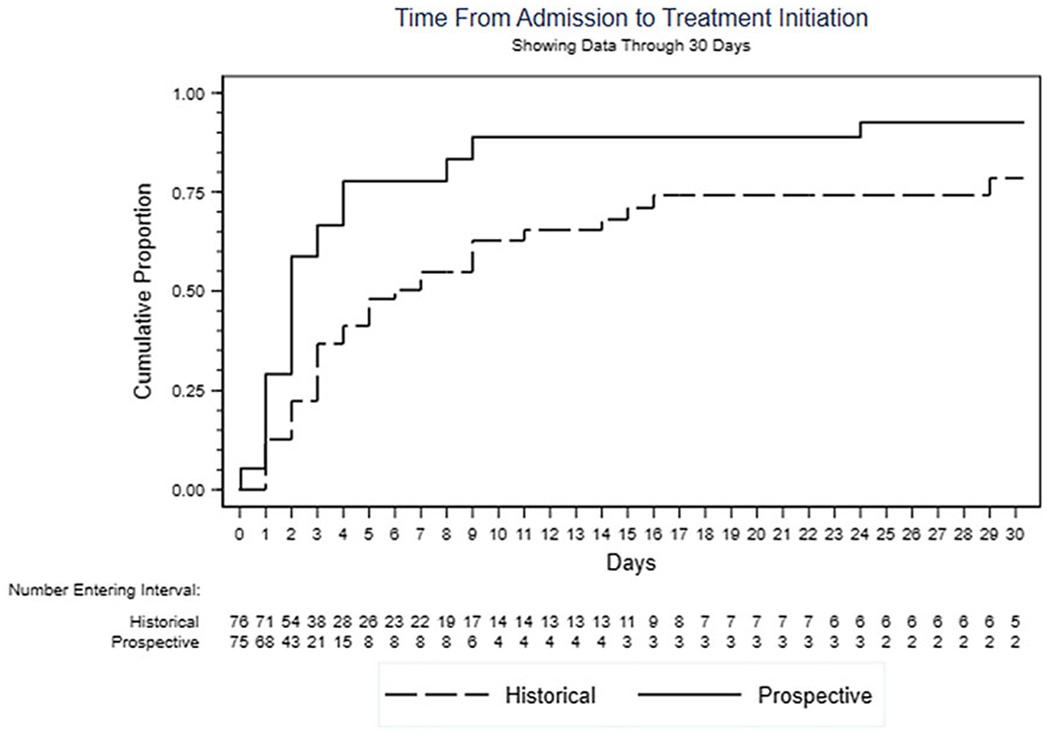
FAST decreases time from hospital admission to start of tuberculosis treatment.

Analyzing the entire period from hospital admission to the start of TB treatment, time to event was shorter under the FAST strategy (HR, 2.11; 95% CI, 1.39–3.21; *P* < .001) (Fig. 2). Overall, 44 (58.7%) patients in the FAST cohort and 22 (28.9%) in the historical cohort received indicated treatment during hospitalization (OR, 3.48; 95% CI, 1.77–6.85; *P* < .001). The FAST cohort patients also experienced fewer days from admission to initiation of indicated treatment (HR, 2.81; 95% CI, 1.67–4.73; *P* = .001). Furthermore, the time to discharge was shorter in the FAST cohort than in the historical control (HR, 1.38; 95% CI, 0.99–1.91; *P* = .058).

## Discussion

During FAST implementation at a general hospital in Lima, Peru, patients with pulmonary TB were more likely to start TB treatment during hospitalization and experience shorter time from admission to treatment than a cohort of historical patients hospitalized prior to FAST implementation. Our findings extend the accumulating body of evidence linking FAST to decreased time to TB treatment by demonstrating that the strategy can accelerate care outside of the specialized TB hospital.^27,30^

Our study uniquely assessed how FAST could be used to enhance indicated treatment, that is, a regimen tailored to DST results and following local guidelines. This distinction is pertinent because prompt delivery of effective treatment based on DST is paramount to interrupting TB transmission.^23,24^ Rapid DST also allows scarce isolation facilities to be prioritized for patients with DR-TB. These effects are particularly important in settings like Peru, where empiric first-line TB therapy cannot be assumed to be effective in newly diagnosed patients given the high prevalence of DR-TB.^1,20^

Universal TB screening during routine care for all hospitalized patients is a key intervention in high TB–incidence settings. In 41 months at HNHU, under the FAST strategy >67,000 patients were screened for symptoms. Screening took place primarily in the emergency department, where the risk for TB transmission is high.^31^ HNHU serves a much broader population of patients than the chest or TB hospitals where FAST was previously pilot tested.^32^ Nevertheless, the number of detected cases at HNHU corresponds to an extremely high TB prevalence: nearly 2,400 of 100,000 symptomatic individuals and >6,000 of 100,000 among those with symptoms who consented to participate. This burden of TB disease is comparable to that found in prisons and other institutional settings in which routine screening for active disease is recommended to reduce transmission and improve outcomes.^33^

The FAST screening protocol at HNHU evolved over the course of the study. The final iteration queried patients about any duration of cough and/or other risk factors for TB. Emerging data, however, demonstrate that TB in asymptomatic patients may also contribute to transmission.^34^ Future research should examine the feasibility and performance characteristics of a FAST strategy that starts with a chest radiograph (irrespective of symptoms) to identify additional individuals with transmissible TB disease.

Xpert testing provided rapid and highly sensitive information about the presence of *Mycobacterium tuberculosis*. The increased sensitivity of Xpert testing led to the diagnosis of TB in 26 FAST patients (35%) in whom smear microscopy was negative. Xpert testing was also likely a prime factor in decreasing the overall time to TB treatment initiation, as has been demonstrated in other settings.^35^

The use of Xpert testing as part of the FAST strategy additionally led to the identification of 18 patients (24%) with RR/MDR-TB in the prospective cohort relative to only 4 (5.3%) with MDR-TB in the historical cohort. There was no reported secular increase in the prevalence of MDR-TB between these contiguous study periods. It is more likely that some patients with RR/MDR-TB were missed in the historical cohort because sensitive, rapid DST was not widely performed. In settings with a high prevalence of RR/MDR-TB, universal Xpert testing for all patients being evaluated for pulmonary TB should be the standard of care to reduce missed RR/MDR-TB diagnoses.

Relative to the historical controls, a higher percentage of patients with pulmonary TB in the FAST cohort received the indicated treatment within 2 days of diagnosis. Although GenoType was used for DST in nearly 60% of the historical cohort, it was not performed in real time; samples were batch-processed, thereby prolonging the interval to results. In contrast, under the FAST strategy, regimens were was informed by near real-time Xpert (and GenoType) results. Thus, it was possible to avoid empiric treatment and to promptly start regimens informed by results from rifampin (and isoniazid) resistance testing. The 2-day benchmark for indicated treatment should be achievable for most patients hospitalized with TB, even for those with DR-TB, given the advent of rapid molecular tests like Xpert to guide regimen composition (especially with development of cartridges that test resistance to isoniazid and fluoroquinolones, as well as rifampin). We suggest treatment initiation within 2 days as a minimum target for patients hospitalized with TB. Future efforts should try to reduce that time even further.

Shortening the time to treatment for patients with TB is critical to decreasing TB transmission by reducing the duration of infectiousness.^36^ In settings with a high burden of MDR-TB, increasing the speed of effective treatment for MDR-TB can lead to decreased transmission of MDR-TB, as previously demonstrated in a FAST pilot test in Russia.^37^ In future research, we will assess the effect of FAST on TB transmission in hospital workers at HNHU.

Although the FAST strategy is conceptually straightforward, successful implementation depends on overcoming practical barriers to coordinated care in a complex hospital environment. The use of trained FAST assistants to facilitate the TB care cascade likely helped to overcome these barriers at HNHU. One potential mechanism may have been the expedited transfer of sputum samples to the laboratory and communication of diagnostic testing results to clinicians. Additionally, FAST assistants referred patients to HNHU physicians for consideration of therapy; if necessary, they also moved medications from the pharmacy to the ward to ensure that treatment was started as soon as possible. We expect that FAST can be adapted to local environments by training existing staff with modest additional inputs from healthcare systems. FAST implementation outside the study conditions has been demonstrated in TB hospitals in Bangladesh and Russia.^32,37^

Our study has several limitations. First, comparing FAST patients to a historical cohort potentially introduces selection bias. Because the historical cohort comprised only patients with positive smear microscopy, we may have underestimated the impact of FAST. If patients with smear-negative TB were included in the historical control, time to TB diagnosis and treatment would likely have been longer because these relied on batched GenoType or mycobacterial culture. Second, the relatively short inclusion period for the historical cohort precluded detection of seasonal variations or other time trend effects. Third, 1,888 eligible patients (60.4%) opted not to participate. Their inclusion would have more than doubled the number of patients tested and/or treated and might have overwhelmed the system, diluting the effects of the FAST implementation. It is also possible, however, that the benefits of the FAST strategy might be more pronounced with a larger intervention sample size. Notably, if the FAST strategy became routine, it would not require consent and participation would be universal, potentially leading to benefits for more patients in the form of faster time to TB diagnosis and treatment.

In conclusion, FAST improves the likelihood and speed of DST-informed treatment for patients hospitalized with TB. This impact is important for TB transmission control because it reduces the time during which hospital transmission can occur.^36^ Faster TB treatment also represents a measurable improvement in quality of care for patients, meeting an urgent need in countries with a high burden of TB.^38^ Broader roll out of the FAST strategy in general hospitals in TB-endemic settings should be considered as part of a comprehensive TB transmission control approach that includes environmental controls and personal protective equipment, making these facilities safer for visitors, staff and patients.

## Figures and Tables

**Table 1.** Diagnostic Testing Utilization and Results During FAST Implementation Compared to Historical Controls

Variable	Prospective (n = 75), No. (%)	Historical (n = 76), No. (%)	*P* Value
Age, mean y	37.1	35.9	.71
Sex, male	45 (60.0)	48 (63.2)	.69
**TB diagnostic test method and results**			
Positive by smear only	8 (10.6)	76 (100)	<.001
Positive by smear and Xpert	41 (54.7)	N/A	
Positive by Xpert only	26 (34.7)	N/A	
**Molecular DST results** ^a^			
No molecular DST result - RR/MDR-TB unknown^b^	1 (1.3)	32 (42.1)	<.001
RR/MDR-TB ruled out by molecular DST	53 (70.7)	40 (52.6)	.004
RR/MDR-TB established by molecular DST	18 (24)	4 (5.3)	.001

Note. TB, tuberculosis; Xpert, Xpert MTB/RIF; DST, drug-susceptibility testing; RR, rifampin resistance; MDR, multidrug resistant (resistance to isoniazid and rifampin).

a Molecular DST for the prospective cohort included Xpert MTB/RIF and/or GenoType MTBDRplus. Molecular DST for the historical cohort was performed by GenoType MTBDRplus alone.

b Includes 1 patient in the prospective cohort who had indeterminant testing for rifampicin resistance by Xpert MTB/RIF.

**Table 2.** Median Time to TB Care Cascade Event at Hospital National Hipolito Unanue^a^

Period	Prospective Median Days (IQR)	Historical Median Days (IQR)	HR (95% CI)	*P* Value
Admission to diagnosis	1 (0–2)	0 (0–1)	0.88 (0.63–1.22)	.46
Admission to diagnosis with DST	1 (1–3)	66 (23–87)	23.71 (10.39–51.11)	<.001
Diagnosis to treatment	1 (0–2)	5 (2–11)	2.54 (1.66–3.89)	<.001
Admission to treatment	2 (1–4)	6 (3–26)	2.11 (1.39–3.21)	<.001
Admission to indicated treatment	4 (2–NR)	NR (6–NR)	2.81 (1.67–4.73)	.001
Admission to discharge	5 (2–12)	6.5 (2–16.5)	1.38 (0.99–1.91)	.058

Note. TB, tuberculosis; DST, drug susceptibility testing; IQR, interquartile ratio; HR, hazard ratio; CI, confidence interval; NR, not reached.

a NR denotes not reached (ie, 50% of participants did not reach the end point).
